# Classic Bronchoscopic Nodularity Sparing the Posterior Wall: A Case of Tracheobronchopathia Osteochondroplastica

**DOI:** 10.7759/cureus.96562

**Published:** 2025-11-11

**Authors:** Shaheen Rizly

**Affiliations:** 1 Medicine, New York Presbyterian Brooklyn Methodist Hospital, Brooklyn, USA

**Keywords:** benign tracheal nodules, bronchoscopy, endobronchial nodules, large airway disease, tracheobronchopathia osteochondroplastica

## Abstract

Tracheobronchopathia osteochondroplastica (TPO) is a rare, benign disorder of the large airways, characterized by multiple submucosal osteocartilaginous nodules, typically sparing the posterior membranous wall. This report describes the case of an 84-year-old female with exertional dyspnea and a diagnosis of TPO confirmed through classic bronchoscopic findings, corroborated by CT imaging and normal pulmonary function tests (PFTs). The report assesses the current understanding of TPO pathophysiology, including recent insights into its molecular mechanisms, the spectrum of conditions that can mimic TPO, common diagnostic challenges and approaches utilizing imaging and bronchoscopy, and management strategies, including rare but potentially serious complications.

## Introduction

Tracheobronchopathia osteochondroplastica (TPO) is an uncommon, benign disorder of the tracheobronchial tree, first described in the 19th century and characterized by the presence of multiple submucosal nodules composed of bone and/or cartilage [1]. These nodules protrude into the airway lumen, most often affecting the anterolateral walls of the trachea and sparing the posterior membranous wall [1]. The etiology and pathogenesis of TPO remain poorly understood; however, recent advances in molecular biology have provided new insights into the role of airway basal stem cells and aberrant signaling pathways [2]. Clinically, TPO is often discovered incidentally or during evaluation for nonspecific respiratory symptoms, and its diagnosis relies on a combination of bronchoscopic, radiologic, and, in selected cases, histopathological findings [3].

TPO is a rare disease, with an estimated prevalence ranging from 0.01% to 0.8%, and approximately 500 cases have been reported worldwide, primarily as case reports or small case series [3]. The differential diagnosis is broad, encompassing both benign and malignant conditions, all of which may present with tracheal wall nodularity. Consequently, identifying TPO is crucial to prevent misdiagnosis, unnecessary biopsies, and inappropriate treatment [4]. Management is typically conservative in mild cases, but rare complications such as severe airway obstruction or massive hemoptysis may require intervention [5]. This report presents a patient with TPO diagnosed based on classic bronchoscopic and imaging findings, reviews the current understanding of TPO, and outlines strategies for its diagnosis and management.

## Case presentation

An 84-year-old female with a medical history notable for hypertension, hyperlipidemia, left total knee arthroplasty, centrilobular emphysema, multiple small pulmonary nodules, and a remote history of tobacco use (one pack per day for 15-20 years, quit 50 years ago), presented to the pulmonary clinic for evaluation of chronic exertional dyspnea. Her oncologic history included proximal descending colon adenocarcinoma, for which she had undergone laparoscopic left hemicolectomy with primary anastomosis two years ago, and a diagnosis of Lynch syndrome.

The patient reported progressive shortness of breath with moderate activity, which had been present for several years, and a new onset of hoarseness for approximately one week. She denied associated fevers, chills, or night sweats. There was no history of recent cough, hemoptysis, chest pain, or unintentional weight loss. She had recently been initiated on fluticasone furoate/umeclidinium/vilanterol (FF/UMEC/VI) inhaler one week before presentation for maintenance therapy of presumed chronic obstructive pulmonary disease (COPD), given her history of tobacco exposure and radiographic evidence of emphysema.

She had not experienced any acute exacerbations or hospitalizations for respiratory symptoms. She had no known history of occupational or environmental exposures, and there was no family history of pulmonary disease. She denied recent travel, contact with sick individuals, or exposure to tuberculosis. On review of systems, she denied orthopnea, paroxysmal nocturnal dyspnea, lower extremity edema, or other symptoms suggestive of heart failure. She had not experienced any recent changes in appetite or gastrointestinal symptoms. Her current medication regimen included antihypertensives, statin therapy, and an FF/UMEC/VI inhaler. She had no known drug allergies.

**Figure 1:**
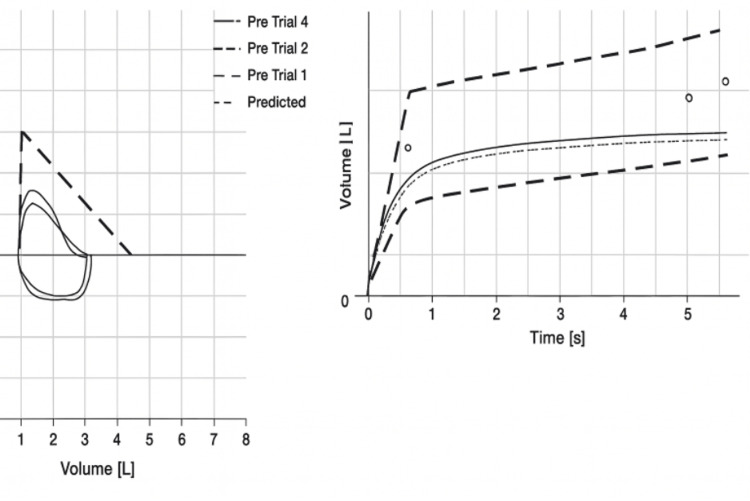
Spirometry and flow volume loop diagrams

Physical examination revealed an elderly, well-appearing female in no acute distress. She was speaking in full sentences without accessory muscle use. Cardiopulmonary examination was unremarkable: lungs were clear to auscultation bilaterally with no wheezing, ronchi, or crackles, and the cardiovascular exam was normal without edema or jugular venous distension. Routine laboratory findings were unrevealing (Table 1). Pulmonary function tests (PFTs) were obtained to objectively assess for obstructive or restrictive physiology in the context of her dyspnea and presumed emphysema. PFTs demonstrated unremarkable spirometry findings except for a mild reduction in FVC, normal FEV1, preserved FEV1/FVC ratio, and supranormal FEF25-75, suggesting the absence of both obstructive and small airway disease (Figure 1, Table 2). Lung volumes and diffusion capacity were not obtained as there was no clinical suspicion of restrictive or diffusion-limiting pathology.

**Table 1:** Routine laboratory results on admission

Test category	Laboratory test	Result	Reference range	Interpretation
Complete blood count	Hemoglobin	13.7 g/dL	13.0 – 17.0 g/dL	Normal
	White blood cell count	7.1 ×10⁹/L	4.0 – 11.0 ×10⁹/L	Normal
	Platelets	245 ×10⁹/L	150 – 450 ×10⁹/L	Normal
Basic metabolic panel	Sodium	139 mmol/L	135 – 145 mmol/L	Normal
	Potassium	4.2 mmol/L	3.5 – 5.1 mmol/L	Normal
	Blood urea nitrogen (BUN)	12 mg/dL	7 – 20 mg/dL	Normal
	Creatinine	0.9 mg/dL	0.6 – 1.3 mg/dL	Normal
Liver function tests	Aspartate aminotransferase (AST)	22 U/L	10 – 40 U/L	Normal
	Alanine aminotransferase (ALT)	26 U/L	7 – 56 U/L	Normal
	Alkaline phosphatase	88 U/L	40 – 130 U/L	Normal
	Total bilirubin	0.6 mg/dL	0.1 – 1.2 mg/dL	Normal
Coagulation profile	Prothrombin time (PT)	12.1 sec	11.0 – 13.5 sec	Normal
	International normalized ratio (INR)	1	0.8 – 1.2	Normal
	Activated partial thromboplastin Time	29 sec	25 – 35 sec	Normal
Inflammatory markers	C-reactive protein (CRP)	2.1 mg/L	<5.0 mg/L	Normal
	Erythrocyte sedimentation rate (ESR)	12 mm/hr	0 – 20 mm/hr (M)	Normal

**Table 2:** Pulmonary function test results at presentation and interpretation Reference values adapted from the American Thoracic Society (ATS)/European Respiratory Society (ERS) guidelines [6] FVC: forced vital capacity; FEV1: forced expiratory volume in 1 second; FEF25–75: mean forced expiratory flow between 25% and 75% of FVC; PEF: peak expiratory flow; FET: forced expiratory time; FIVC: forced inspiratory vital capacity; PIF: peak inspiratory flow

Parameter	Predicted	LLN	Best	Trial 1	Trial 2	Trial 3	% Predicted	Interpretation
FVC (L)	3.07	2.27	2.45	2.13	2.58	2.45	80	Mild reduction, age-related
FEV1 (L)	2.25	1.61	1.93	1.76	1.92	1.93	84	Normal
FEV1/FVC	0.729	0.631	0.787	0.824	0.686	0.787	103	No obstruction
FEF25–75 (L/s)	1.50	0.59	1.76	2.24	1.49	1.78	119	Normal/supranormal
PEF (L/s)	5.20	3.20	3.86	3.86	3.21	3.22	74	Mildly reduced
FET (s)	–	–	6.6	6.2	7.0	6.6	–	Within the expected range
FIVC (L)	3.07	2.27	2.58	2.27	2.49	2.58	84	Normal
PIF (L/s)	–	–	2.43	2.43	2.13	2.36	–	Adequate inspiratory flow

**Figure 2:**
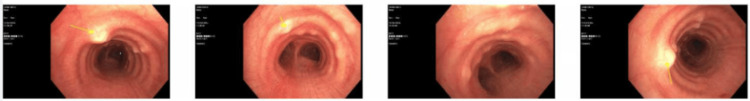
Bronchoscopic image demonstrating multiple, hard, whitish submucosal nodules protruding into the tracheal lumen, with sparing of the posterior wall (as demonstrated with yellow arrows)

Given this discrepancy, a CT scan of the chest was ordered to evaluate for other structural causes of dyspnea or airway symptoms. CT revealed mild centrilobular emphysema and subtle nodularity along the anterior tracheal wall, sparing the posterior membranous wall, raising concern for TPO. To further evaluate the extent and nature of the airway findings, a flexible diagnostic bronchoscopy was performed, which revealed multiple, hard, whitish submucosal nodules along the cartilaginous portions of the trachea and mainstem bronchi, again sparing the posterior wall (see Figure 2).

The carina was sharp, and no endobronchial lesions or secretions were observed. The diagnosis of TPO was established based on characteristic bronchoscopic and imaging findings, without the need for endobronchial biopsy. The disease was determined to be mild in severity, with no evidence of luminal compromise or active infection. The patient was managed conservatively with education and reassurance about the benign nature of the condition. Her inhaled therapy was continued due to reported symptomatic improvement. No further follow-up was planned unless her symptoms worsened.

## Discussion

Pathophysiology

The pathogenesis of TPO has historically been attributed to chronic inflammation, mechanical irritation, and metaplastic processes [1]. Recent molecular studies have revealed a central role for airway basal stem cell dysfunction and aberrant transforming growth factor-beta (TGFβ)- bone morphogenetic protein (BMP) signaling. Hong et al. demonstrated that basal cells in TPO act as a repository for inflammatory and TGFβ-BMP signals, driving both epithelial squamous metaplasia and submucosal osteochondrogenesis via extracellular signaling and matrix remodeling. This results in the formation of mature bone and cartilage within the airway wall, accompanied by chronic inflammation and extracellular matrix remodeling [2].

These findings provide a link explaining the mechanisms between chronic inflammation, aberrant stem cell function, and the characteristic histopathology of TPO. Other studies have highlighted the association of TPO with chronic airway inflammation, comorbid conditions such as COPD and gastroesophageal reflux disease (GERD), and, in rare cases, interstitial lung disease, further supporting the role of inflammatory and profibrotic mediators in disease pathogenesis [3,7,8]. Recurrent respiratory infections are also a recognized complication of TPO, and the presence of airway nodularity and luminal narrowing may predispose to infection, likely by impairing normal airway defenses and clearance mechanisms [1,9].

Diagnostic criteria and approach

The diagnosis of TPO is established primarily based on classic bronchoscopic findings, supported by imaging. The hallmark bronchoscopic appearance is that of multiple, hard, whitish or yellowish submucosal nodules protruding into the anterolateral walls of the trachea and proximal bronchi, with sparing of the posterior membranous wall [1,3,10]. The submucosal nodules in TPO originate from the cartilaginous rings of the trachea, which are present only in the anterior and lateral walls, while the posterior wall is composed of membranous tissue and lacks cartilage. As a result, the pathological process of cartilage and bone formation does not involve the posterior membranous wall, leading to its characteristic sparing in TPO [1,9,10].

CT imaging typically reveals submucosal nodules with or without calcification, again sparing the posterior wall [3,9]. Histopathological confirmation is reserved for atypical or uncertain cases, or when alternative diagnoses such as malignancy or amyloidosis are suspected [3,11]. The specific histological and pathological findings in TPO are submucosal nodules composed of mature cartilage and bone (ossification), often with associated calcium deposits, located beneath an intact or metaplastic respiratory epithelium [9]. Epithelial squamous metaplasia, heterotopic bone formation, abnormal cartilage proliferation, and chronic inflammation may also be present [2]. These nodules protrude into the airway lumen from the anterolateral tracheal and bronchial walls, and histopathology confirms the diagnosis by demonstrating these characteristic features [10].

Differential diagnosis

The differential diagnosis for tracheal nodularity and submucosal lesions in elderly patients is broad and includes tracheobronchial amyloidosis, relapsing polychondritis, granulomatosis with polyangiitis, sarcoidosis, recurrent respiratory papillomatosis, broncholithiasis, and both benign and malignant neoplasms [4,12]. TPO is distinguished by the distribution and character of the nodules (anterolateral wall, sparing the posterior wall, hard and sessile), the absence of systemic features, and the lack of cytologic atypia or granulomatous inflammation on histopathology [1,3,10]. Amyloidosis, for example, often involves the posterior wall and presents as friable, waxy plaques, while relapsing polychondritis is associated with systemic cartilage inflammation and destruction [12]. Neoplasms are typically solitary, irregular, and may show invasive features [4]. These distinctions are summarized in Table 3.

**Table 3:** Diagnostic criteria and distinguishing features of TPO and its mimickers* ^*^[4] TPO: tracheobronchopathia osteochondroplastica

Condition	Bronchoscopic features	Imaging features	Distinguishing features
TPO	Hard, whitish nodules, anterolateral, sparing the posterior wall	Nodular/calcified thickening, sparing the posterior wall	Multiple, submucosal, no atypia
Amyloidosis	Waxy, friable, yellowish plaques, often posterior wall	Irregular, sometimes calcified, circumferential	Amyloid deposits, Congo red positive
Relapsing polychondritis	Diffuse inflammation, malacia	Diffuse/segmental thickening	Systemic features, cartilage destruction
Neoplasms	Solitary/multifocal, irregular, ulcerated	Focal/circumferential thickening, mass effect	Solitary, irregular, may not spare the posterior wall
Granulomatous diseases	Nodular, plaque-like, variable wall involvement	Wall thickening, nodularity, parenchymal findings	Granulomatous inflammation, no bone/cartilage

Diagnostic pitfalls and sources of misdiagnosis

TPO is frequently underdiagnosed or misdiagnosed due to its nonspecific clinical presentation and overlap with more common airway diseases such as asthma, COPD, or chronic bronchitis [3,10,11,13]. Misinterpretation of imaging, failure to perform or interpret bronchoscopy, and confusion with other causes of tracheal nodularity are common [1,4,14]. Overreliance on histopathology, particularly when biopsy samples are superficial or not representative, can also lead to misdiagnosis [1,3,10,14]. Therefore, recognizing the characteristic bronchoscopic and imaging features of TPO and considering the diagnosis in patients with unexplained chronic airway symptoms is essential to avoid misdiagnosis [7,11,13].

PFT interpretation

In this patient, spirometry revealed no evidence of obstruction or restriction, and the mild reduction in FVC was consistent with age-related decline. The absence of small-airway disease was supported by a supranormal FEF₂₅-₇₅. These findings are typical in mild TPO, where the disease is often limited to the large airways and does not produce significant airflow limitation [3,5]. The lack of correlation between symptoms and PFTs reinforces the importance of direct airway visualization in diagnosing TPO.

Although no published studies have specifically applied computational fluid dynamics (CFD) or airflow modeling to TPO, CFD analyses of focal tracheal stenosis and laryngotracheal pathology have shown that discrete intraluminal protrusions can generate turbulent eddies, elevate local wall shear stress, and reduce peak expiratory flow rates, even when overall airway cross-sectional area is not critically reduced [15,16]. By analogy, these findings are likely applicable to TPO, particularly in cases where clustered submucosal ossified nodules create irregular, focal narrowing or disrupt laminar flow, which may explain exertional dyspnea that is out of proportion to spirometric findings.

Management strategies and follow-up

The management of TPO is guided by the severity of symptoms and the degree of airway involvement. In mild or asymptomatic cases, as in this patient, conservative management is recommended, with a focus on patient education, avoidance of airway irritants, and treatment of respiratory infections [3,5,8,9]. Inhaled corticosteroids may be considered in selected cases with early-stage disease or coexisting airway inflammation, but there is limited evidence for their efficacy [8,11]. Interventional bronchoscopic procedures, such as mechanical debulking or stent placement, are reserved for patients with severe symptoms, significant airway obstruction, or complications such as recurrent pneumonia [11,14,17]. There is no indication for routine intervention in mild cases.

There are no established guidelines or structured protocols for follow-up in TPO, largely because of its indolent course and minimal clinical consequences in most cases. This aligns with the observation that TPO is generally benign and stable, with most patients not requiring intervention or routine surveillance unless symptoms or clinical status change [3,5]. However, it is reasonable to perform annual clinical assessments and to repeat PFTs if new or worsening symptoms develop. Routine serial PFTs are not necessary in the absence of clinical change. Repeat bronchoscopy or imaging should be reserved for cases with new or progressive symptoms or suspicion of disease progression. Management of comorbidities such as COPD or GERD is important, as these can coexist and may affect symptom severity or complication risk [3].

Prognosis and complications

The long-term prognosis for patients with mild TPO is favorable, with most cases demonstrating a benign, indolent course and minimal risk of significant progression or life-threatening complications [3,5,8]. The risk of severe airway obstruction, massive hemoptysis, or recurrent infection is low in mild cases, but closer monitoring is recommended in patients with extensive disease, baseline airway narrowing, or severe comorbidities [3]. Rare but serious complications, such as massive hemoptysis or critical airway obstruction, require prompt, multidisciplinary management, including airway protection, and, if needed, bronchial artery embolization or interventional bronchoscopy [11,14].

Perioperative airway management

Patients with TPO are at increased risk for difficult airway management during surgery due to submucosal nodularity and potential tracheal narrowing [18]. The American Society of Anesthesiologists recommends a preformulated strategy for managing anticipated difficult airways, including consideration of awake fiberoptic intubation, use of smaller, well-lubricated endotracheal tubes, and preparation for emergency invasive airway access [19]. As such, preoperative review of bronchoscopy and imaging findings and multidisciplinary planning is recommended [19].

Quality of life and patient-reported outcomes

The impact of TPO on patient-reported quality of life and functional status is generally mild in most cases, with the majority of patients experiencing stable symptoms and minimal impairment [3,5]. There are no validated, disease-specific patient-reported outcome measures (PROMs) for TPO, and no case series in the last decade have systematically assessed quality of life using standardized instruments. Generic instruments such as the 36-Item Short Form Survey Instrument (SF-36), EuroQol-5D (EQ-5D), and Patient-Reported Outcomes Measurement Information System (PROMIS) are recommended for use in rare airway diseases, such as bronchiectasis and idiopathic pulmonary fibrosis [20,21]. Such PROMs could potentially be applied to TPO, but have not been systematically used in published TPO cohorts, given their limitations in sensitivity and specificity for rare conditions. The lack of systematic PROMs data in TPO represents a gap in the literature and an opportunity for future research.

Future directions

To date, no multicenter registries or prospective natural history cohorts exist for TPO. Published studies are limited to single-center case series and retrospective reviews, and there is no infrastructure for systematic long-term outcome tracking or interval surveillance. The formation of a multicenter or multinational TPO registry would enable studies to better define progression rates and risk factors for complications.

## Conclusions

This report highlights the classic presentation and benign course of mild TPO, a rare disorder of the large airways. The diagnosis was made based on characteristic bronchoscopic and imaging findings, with normal pulmonary function tests, and without histopathological confirmation. While this could be considered a diagnostic limitation, it is justified given the patient’s stability and the typical clinical features of TPO. Recent advances in the understanding of TPO pathogenesis emphasize the role of airway basal stem cell dysfunction and chronic inflammation. The differential diagnosis is broad, but TPO can be distinguished by its radiologic and bronchoscopic features. Management is conservative in mild cases, with a focus on patient education and periodic clinical follow-up. The prognosis is excellent, and the risk of progression or serious complications is low. Awareness of TPO and its mimickers is important to avoid diagnostic pitfalls and ensure appropriate management.
